# Y-Chromosome Genetic Analysis of Modern Polish Population

**DOI:** 10.3389/fgene.2020.567309

**Published:** 2020-10-23

**Authors:** Łukasz Grochowalski, Justyna Jarczak, Maria Urbanowicz, Marcin Słomka, Maria Szargut, Paulina Borówka, Marta Sobalska-Kwapis, Błażej Marciniak, Andrzej Ossowski, Wiesław Lorkiewicz, Dominik Strapagiel

**Affiliations:** ^1^Biobank Lab, Department of Molecular Biophysics, Faculty of Biology and Environmental Protection, University of Lodz, Łódź, Poland; ^2^BBMRI.pl Consortium, Łódź, Poland; ^3^Department of Forensic Genetics, Pomeranian Medical University in Szczecin, Szczecin, Poland; ^4^The Polish Genetic Database of Totalitarianism Victims, Szczecin, Poland; ^5^Department of Anthropology, Faculty of Biology and Environmental Protection, University of Lodz, Łódź, Poland

**Keywords:** Y-chromosome, haplogroups, Polish population, regions of Poland, microarray analysis, SNPs

## Abstract

The study presents a full analysis of the Y-chromosome variability of the modern male Polish population. It is the first study of the Polish population to be conducted with such a large set of data (2,705 individuals), which includes genetic information from inhabitants of all voivodeships, i.e., the first administrative level, in the country and the vast majority of its counties, i.e., the second level. In addition, the available data were divided into clusters corresponding to more natural geographic regions. Genetic analysis included the estimation of *F*_ST_ distances, the visualization with the use of multidimensional scaling plots and analysis of molecular variance. Y-chromosome binary haplogroups were classified and visualized with the use of interpolation maps. Results showed that the level of differentiation within Polish population is quite low, but some differences were indicated. It was confirmed that the Polish population is characterized by a high degree of homogeneity, with only slight genetic differences being observed at the regional level. The use of regional clustering as an alternative to counties and voivodeships provided a more detailed view of the genetic structure of the population. Those regional differences identified in the present study highlighted the need for additional division of the population by cultural and ethnic criteria in such studies rather than just by geographical or administrative regionalization.

## Introduction

The structure and variability of the modern Polish population have arisen as a result of the demographic and political changes that have formed the populations of this part of Europe. Poland was first regarded as a nation with the beginning of the Piast state (the so-called first Polish state) in the 10th century AD. The early history of the inhabitants of the land between the Oder and Bug rivers is inseparably connected with the discussion on the ethnogenesis of the Slavs. According to the autochthonous hypothesis, the Slavs developed and lived in the Oder and Vistula basins, and their roots in this area extend back to 1,200 to 1,000 years BC. In contrast, the allochthonous theory assumes that the Slavs arrived in this area between the fifth and sixth century CE from the Upper Dnieper basin, an area believed to be their cradle (Trzeciecki, 2016). This 100-year-old discussion has recently been joined by anthropologists and geneticists studying modern mtDNA and Y-chromosome polymorphisms (Malyarchuk et al., 2002, 2008; Branicki et al., 2005; Grzybowski et al., 2007; Rebala et al., 2007, 2013; Wozniak et al., 2010; Mielnik-Sikorska et al., 2013a) and recently also ancient DNA (Juras et al., 2014).

Polish modern history, especially during the last 200 years, was rich in dramatic events such as wars, occupations, borders shifting, and political migrations. However, the greatest influence for the shaping of modern demographic situation had consequences of World War II (WWII). Until that time, population of Poland was an ethnic, religious, and linguistic mosaic, in which people have coexisted together for centuries [native Polish in 1939-65.5% population (Polish Ministry of Information, 1941)]. The final number of victims during WWII was estimated at more than 6 million of Polish citizens (Polish War Reparations Bureau, 1947), which meant greater than 17% of prewar population of Poland (Polish Ministry of Information, 1941). Because of hostilities, young men constituted a large part of this number whose death resulted in significant depletion of gene pool (Diepenbroek et al., 2019).

Furthermore, the borders of Poland have been radically shifted, which triggered significant demographic changes such as mass resettlements and human migrations. Therefore, millions of people of different ethnicity were suddenly forced to leave their immemorial residence in mass migrations (Eberhardt, 2000). In years 1944–1948, from lands that belonged to Poland before the WWII and have been incorporated to Soviet Republics, around 800,000 Polish people have been officially resettled from Ukrainian SSR (Kersten, 1974; Czerniakiewicz, 1987), which means as much as 96% people registered there for transfer (Piesowicz, 1988). The official migrants were resettled to area between Upper and Lower Silesia (Hryciuk et al., 2008). From the Byelorussian SSR, around 300,000 of Polish people have been resettled (33.5% registered for transfer) (Kersten, 1974; Czerniakiewicz, 1987; Piesowicz, 1988) to Lower Silesia, western part of Greater Poland, Lubusz, Szczecin in West Pomerania, and Gdańsk in Pomerania (Hryciuk et al., 2008). From Lithuanian SSR, around 200,000 of Polish people have been resettled (51.5% registered for transfer) (Kersten, 1974; Czerniakiewicz, 1987) to Warmian–Mazurian, Pomerania, and some of them to Lower Silesia (Hryciuk et al., 2008). Moreover, around 250,000 of Polish people have been also officially resettled from the Soviet Union (Kersten, 1974) (Supplementary Figure S1). About 3 million people also moved there from the rest of Polish territory, comparing almost 1.2 million of native Polish who have already lived in Upper Silesia and Warmian–Mazurian as the indigenous (Kosiński, 1960; Eberhardt, 2000). Furthermore, at the same time almost 2 million Polish people returned to Poland from Western Europe (Kersten, 1974) (Supplementary Figure S2). In years 1955–1959, the next wave of resettlements took place, and 250,000 native Polish have been displaced from the Soviet republics to the new western Polish lands (Latuch, 1994) (Supplementary Figure S1). Other ethnic populations have been displaced in the same way: several millions of Germans moved from new Polish lands to Germany and majority from around 700,000 indigenous Ruthenians and Ukrainians from Subcarpathian were resettled to Ukrainian SSR and 140,000 in operation “Wisła” forcely moved to Lower Silesia, West Pomerania, and Warmian–Mazurian (Eberhardt, 2000) (Supplementary Figure S2).

In summary, in Poland within the past 80 years, more than 11 million people of both Polish and non-Polish descendance have been moved either to or from the country (Ploski et al., 2002). The genetic structure of the country has changed between the prewar and postwar period dramatically (Rebala et al., 2013; Diepenbroek et al., 2019).

Modern population studies are often based on genome-wide analysis studies, most commonly employing single-nucleotide polymorphism (SNP) microarray technology; this approach is capable of identifying disease-related or trait-related variants and is essential for the advancement of personalized or forensic medicine (Tam et al., 2019). However, analysis of the SNPs related with an allosome locus can also be of great value in anthropological and forensic research, as they appear to carry key information about the genetic diversity of a certain population. Knowledge of the phylogenies of the paternally inherited portion of the non-combining region of chromosome Y (NRY) can be acquired by examining the patterns of Y-short tandem repeats (Y-STR); these are subject to a higher mutation rate and thus demonstrate higher typing resolution than the more slowly evolving Y-chromosomal biallelic polymorphisms (Rosser et al., 2000; Gill et al., 2001).

Previous studies tracing paternal lineages and kinship in different parts of the country have analyzed Y-STR haplotype and allele frequencies of Polish men (Pepinski et al., 2004b; Rebala and Szczerkowska, 2004; Wozniak et al., 2007; Soltyszewski et al., 2008; Wolanska-Nowak et al., 2009), as well as studies performed on the representatives of selected cities (Ploski et al., 2002; Kayser et al., 2005; Rebala and Szczerkowska, 2005), and among ethnic groups (Rebala et al., 2007, 2013; Janica et al., 2008), minorities, and residents (Pepinski et al., 2004c, 2005a,b; Janica et al., 2006). These studies have typically employed residual polymerase chain reaction (PCR)–based Y-chromosomal biallelic polymorphism estimation (Rosser et al., 2000), autosomal (Behar et al., 2013), and whole-genome approaches (Lao et al., 2008).

Our study presents a full analysis of the Y-chromosome variability of the modern male Polish population. It is the first study of the Polish population to be conducted with such a large set of data (2,705 individuals), which includes genetic information from inhabitants of all voivodeships, i.e., the first administrative level, in the country and the vast majority of its counties, i.e., the second level. In addition, the available data were divided into clusters corresponding to more natural geographic regions. The obtained results, as yet unpublished, estimate the missing genetic variability of the modern Polish population and examine the genetic relationships between its members, allowing researchers to shed light on the historical, demographic, and social changes that have occurred during the turbulent history of the country. They represent an excellent complement to earlier mtDNA studies on the diversity of the Polish population (Jarczak et al., 2019).

## Materials and Methods

### Subjects

Adult participants were recruited between 2010 and 2012 under the TESTOPLEK project based on general Polish population—POPULOUS collection of 10,000 saliva samples, derived from female and male attendees, completed with individual in-depth interview based on questionnaires. These recorded their place of residence, together with various other questions about the origin or ancestry of parents and grandparents. Saliva samples were collected up to 2016 and collectively have been included to POPULOUS collection at the Biobank Lab of the Department of Molecular Biophysics of the University of Lodz (Strapagiel et al., 2016; Dobrowolska et al., 2019), which is currently registered in Directory (v. 4.0) of BBMRI-ERIC consortium under bbmri-eric:ID:PL_BLUL:collection:POPULOUS_BLUL registration number. Approval for this study was obtained from the University of Lodz Ethics Review Board. All procedures were performed in accordance with the Declaration of Helsinki (ethical principles for medical research involving human subjects).

Finally, a group comprising 2,705 adult male inhabitants of all 16 Polish voivodeships was assembled for the present study. These participants were found to represent 337 of 380 counties (in Polish: *powiaty*). The regional data were assembled into 40 clusters, thus providing a high-resolution overview of the diversity of modern-day male Polish population (Supplementary Figure S3).

### Clustering and Visualization

Cluster formation allowed data from counties with low sample sizes to be merged, to provide a greater density of points than analysis based on voivodeships alone. The data from the counties were merged into 40 clusters using the K-means method (Jarczak et al., 2019).

Clustering was carried out using Python (v.3.7.4) with Scikit-learn package (Pedregosa et al., 2011). The approach resulted in the formation of a number of regions, whose lowest cluster size was 30, and the most numerous was 301. The list of counties and their resulting clusters can be found in Supplementary Table S1.

The geographical representation of the haplogroup frequencies was performed using QGIS (v.2.18.16). Surface interpolation was carried out using the Inverse Distance Weighted method on a valid administrative map of Poland downloaded from the Geodesic and Cartographic Documentation Center website. The longitude and latitude of the counties were obtained with the Google Maps Api.

### Sampling and Genotyping

Saliva was collected from each individual using Oragene OG-500 DNA storage probes. Genomic DNA was manually extracted with PrepitL2P^®^ (PD-PR-052, DNA Genotek, Canada), and the samples were genotyped using Infinium HTS Human Core Exome PLUS microarrays (Illumina, Inc., San Diego, CA, United States), according to the manufacturer’s protocol. Quality control of obtained results was performed by examining raw fluorescence intensities in GenomeStudio (v.2011.1) with Genotyping Module (v.1.9.4) (Illumina, Inc.); all samples met the criteria, demonstrating a call rate greater than 0.98 with the 10% GenCall parameter above 0.4. A total of 1,755 SNPs (Supplementary Table S2) located on the Y-chromosome passed QC and were included in the analysis. StrandScript (Wang et al., 2017) was used to correct strand orientation. The full set of data from genotyping can be found at the European Genotype Archive—the accession number for the Y chromosome microarray data of Polish population reported in this article is EGAS00001004111.

### Bioinformatics Analysis

Genetic variation between, and within, voivodeships and clusters was quantified by analysis of molecular variance (AMOVA) using Arlequin (v.3.5) (Excoffier and Lischer, 2010). Arlequin was also used to calculate pairwise genetic distance (*F*_ST_) for clusters and voivodeships based on the obtained Y-SNP data (*n* = 1,755 SNPs). The statistical significance of the Arlequin analysis was assessed using 10,000 permutations. The pairwise genetic distances were visualized by multidimensional scaling (MDS) analysis using the cmdscale function in R (v.3.4.2).

yHaplo (v.1.0.19) (Poznik, 2016) performed Y-SNP binary haplogroup assignments on 496 informative SNPs. Haplogroup frequencies were calculated for voivodeships and clusters. Links to all web resources mentioned in the text are listed in Appendix A.

## Results

A total of 2,705 unrelated males from the Polish population with place of residence were included in the study. The list of typed haplogroup for each sample is included in Supplementary Table S3. The analysis of allele distribution among the studied samples revealed 12 different haplogroups, of which R was divided into subhaplogroups R1a and R1b for better resolution (Table 1).

**TABLE 1:** Main haplogroups and selected subhaplogroups frequencies for Polish population including division into voivodeships (*n* = 2,705).

**Voivodeship**	**Haplogroups**												**Number of samples**
	**C**	**E**	**G**	**H**	**I**	**J**	**N**	**O**	**Q**	**R1a**	**R1b**	**T**	
Greater Poland	0.00	5.38	1.43	0.00	16.13	3.58	2.15	0.00	0.00	56.99	14.34	0.00	279
Holy Cross	0.00	5.71	0.00	0.00	14.29	2.86	8.57	0.00	5.71	51.43	11.43	0.00	35
Kuyavian–Pomeranian	0.00	2.11	0.70	0.70	11.27	4.93	2.82	0.00	0.00	63.38	14.08	0.00	142
Lesser Poland	0.00	6.04	2.01	0.00	15.44	4.03	2.68	0.00	0.00	54.36	14.77	0,67	149
Lodz	0.88	1.77	0.00	0.00	7.96	0.88	1.77	0.00	0.00	68.14	18.58	0,00	113
Lower Silesia	0.65	3.25	1.95	0.00	23.38	4,55	3.90	0.00	0.00	53.90	8,44	0.00	154
Lublin	0.51	4.04	0.51	0.00	15.66	2.53	4.55	0.00	0,51	62.12	8.59	1.01	198
Lubusz	0.00	1.89	1.89	0.00	25.47	0.94	3.77	0.00	0.00	53.77	12.26	0.00	106
Mazovia	0.00	3,80	0.84	0.00	16.03	2.95	3.38	0.00	0.42	61.18	11.39	0.00	237
Opole	0.00	1.49	2.99	0.00	14.93	5.97	4.48	0.00	2.99	53.73	13.43	0.00	67
Podlaskie	0.00	1.82	0.91	0.00	19.09	0.00	14.55	0.00	0.00	53.64	9.09	0.91	110
Pomeranian	0.00	3.45	0.99	0.00	16.26	0.99	5.91	0.00	0.00	56.16	16.26	0.00	203
Silesia	0.22	4.09	1.51	0.00	15.05	4.09	3.87	0.22	0.22	52.04	18.49	0.22	465
Subcarpathian	0.48	4.33	0.96	0.00	12.50	4.81	2.40	0.00	0.00	57.69	16.83	0.00	208
Warmian–Mazurian	0.00	4.24	0.85	0.00	14.41	4.24	6.78	0.00	0.00	54.24	15.25	0.00	118
West Pomeranian	0.00	4.96	1.65	0.00	14.88	1.65	6.61	0.00	0.00	59.50	10.74	0.00	121
Total	0.18	3.84	1.22	0.04	15.71	3.22	4.29	0.04	0.26	56.93	14.09	0.18	2,705

The most frequent Y-SNP binary haplogroups in all analyzed samples were found to be R (71.02%), I (15.71%), N (4.29%), E (3.84%), J (3.22%), and G (1.22%). The total contribution of the others, *viz.* Q, C, T, H, and O, totaled less than 1% (0.70%), and each comprised only individual samples (Table 1).

The samples were divided to visualize the distribution of haplogroups according to voivodeship. Most were characterized by the presence of six or seven haplogroups (hgs), with only Silesia (10 hgs) and Lublin (9 hgs) being more diverse. While in Silesia this high number may be attributed to the higher number of samples recorded, Lublin, with one less haplogroup identified, recorded a similar number of samples to the other voivodeships. Additionally, most of the voivodeships did not differ with regard to the number of haplogroups, which suggests the population is highly homogeneous (Table 1).

In all voivodeships, hg R was the most common, with the highest frequency observed in the Lodz voivodeship (86.72%) and lowest in Lower Silesia (62.34%) (Table 1). Interestingly, Lodz is represented almost only by haplogroups R and I, accounting for 93.80% of the samples.

**FIGURE 1:**
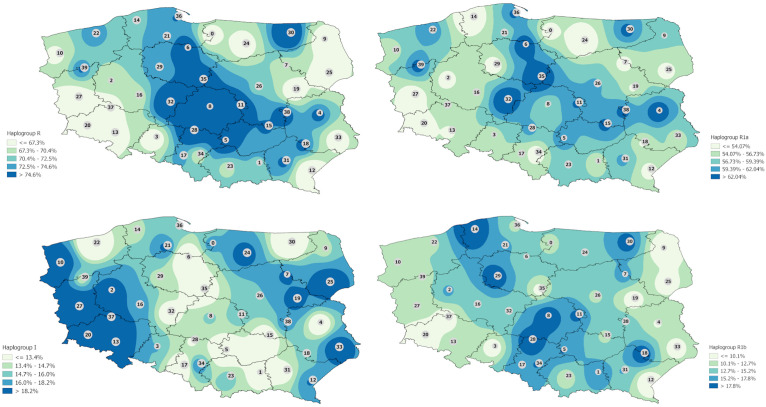
Interpolation maps for the two main haplogroups (R and I) with the division (in case of hg R) into subhaplogroups R1a and R1b observed in the Polish population.

A deeper investigation of haplogroup distribution was carried out based on the clusters. Haplogroup R is unevenly distributed in Polish population with the central part of the country marked by the highest frequencies (Figure 1). When hg R was divided into subhaplogroups, one can see that R1a is distributed mostly in the center part of Poland with a few regions in the west and east of the country. R1b is most widely distributed on the territory of Poland, reaching farther east and west (Figure 1).

**FIGURE 2:**
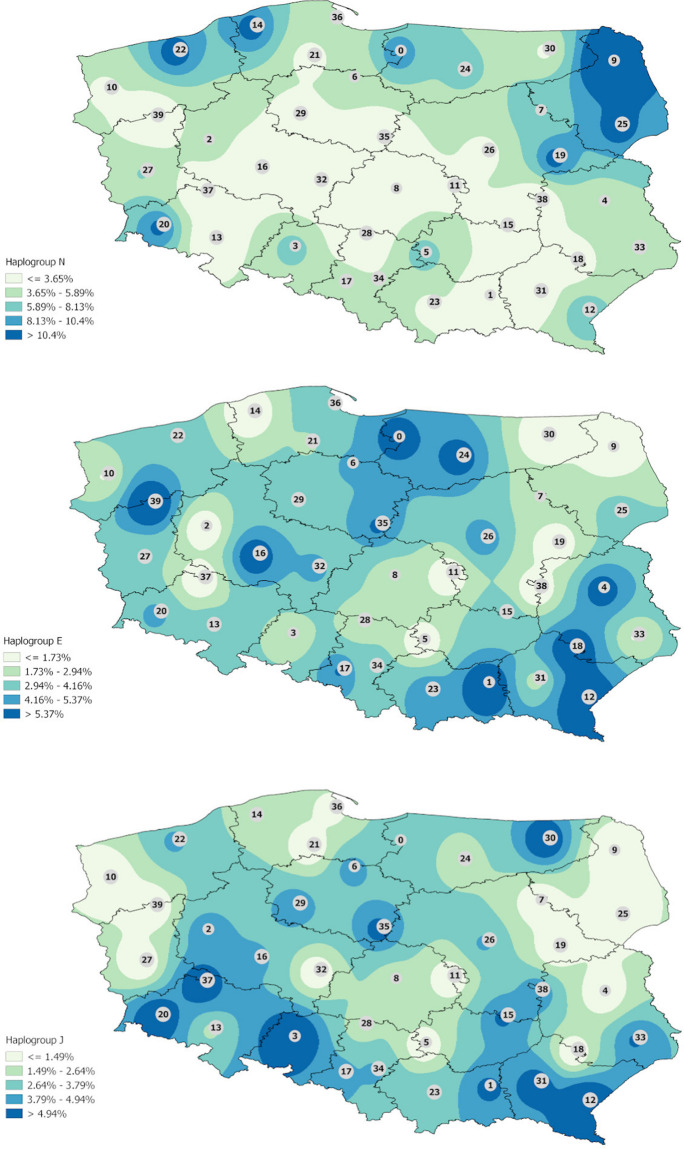
Interpolation maps for the other main haplogroups (N, E and J) observed in the Polish population.

Interpolation map of haplogroup I shows that it is more evenly represented in the Polish population but some trends are indicated. The highest frequencies are observed in western Poland and in some regions of eastern Poland mostly in Podlaskie and Lublin voivodeships but reaching also eastern parts of Mazovia, western parts of Warmian–Mazurian, and almost all Subcarpathian (Figure 1). Haplogroup N is observed mostly in all Podlaskie voivodeship. In the case of haplogroups E and J, the differences are not so highlighted, and a much greater diversity of frequencies is observed (Figure 2).

The maps in Figures 1, 2 present an interpolated distribution of the seven most frequent haplogroups in the Polish population.

### Genetic Differences (*F*_ST_)

To identify changes in genetic distance across the population, voivodeships and clusters were compared by the *F*_ST_ metric, which ranged from 0.0001 to 0.09123, depending on the tested voivodeship (Supplementary Table S4). The highest *F*_ST_ values were observed between Lodz and Lower Silesia (*F*_ST_ = 0.09123; *p* < 0.00001), as well as between Lodz and Podlaskie (*F*_ST_ = 0.085; *p* < 0.00001) (Supplementary Table S4 and Supplementary Figure S4). The results identified Lodz as an outlier, being significantly different to the 14 other voivodeships. Lower Silesia demonstrated the second highest number of statistically significant *F*_ST_ values. Only the Lodz and the Kuyavian–Pomeranian voivodeship pair demonstrated no differences.

**FIGURE 3:**
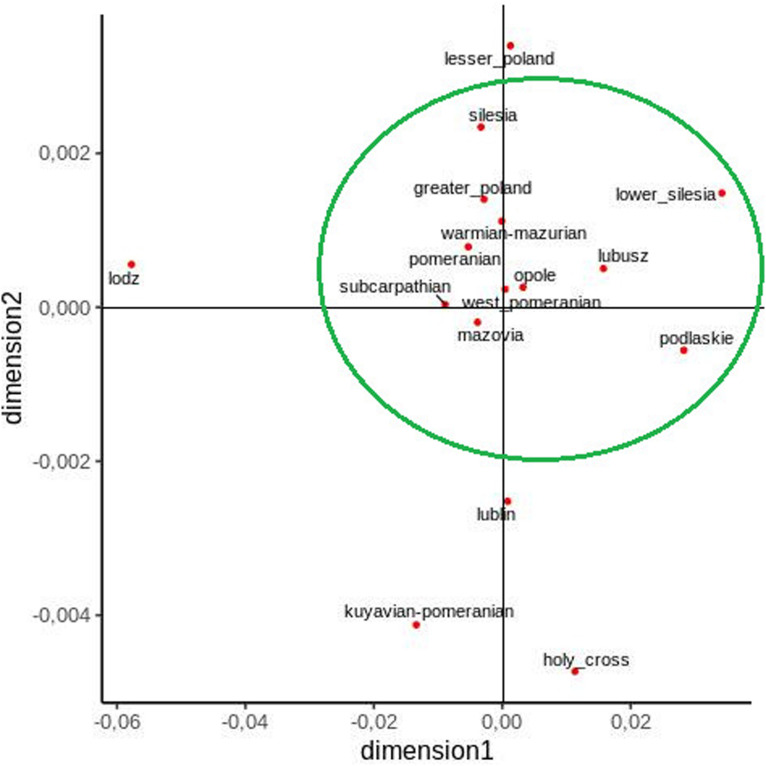
Two-dimensional MDS plot of Polish voivodeship populations based on pairwise F_ST_ values.

Furthermore, an MDS plot, constructed on the basis of pairwise *F*_ST_ values, clearly shows that most voivodeships form a compact group and that the Lodz, Lublin, Kuyavian–Pomeranian, and Holy Cross voivodeships lie outside them (Figure 3).

The paired *F*_*ST*_ analysis performed for clusters returned values ranging from −0.018 to 0.192 (Supplementary Table S5). The highest *F*_*ST*_ estimates were identified between clusters **20** (Lower Silesia—area of Jelenia G ra and Zgorzelec) and **30** (Warmian–Mazurian—area of Giżycko, Ełk, Gołdap) (*F*_*ST*_ = 0.10778, *p* = 0.01562); between clusters **20** and **32** (Greater Poland—Konin, Kalisz, and Sieradz counties) (*F*_*ST*_ = 0.10776; *p* = 0.00098), and between **20** and **28** (a cluster on the border of Silesia, Lodz, and Opole) (*F*_*ST*_ = 0.10692; *p* = 0.00488) (Supplementary Figure S5 and Supplementary Table S5). Interestingly, clusters **20** and **12** (Subcarpathian region including Przemyśl, Sanok, and the Bieszczady mountains) demonstrated the same relations with clusters **30**, **28**, and **32** (*F*_*ST*_ = 0.09196; *F*_*ST*_ = 0.09144; *F*_*ST*_ = 0.09085, respectively *p* = 0.01074; *p* = 0.00781; *p* = 0.00293). In addition, **20** and **12** did not demonstrate significant differences in the number of estimates, despite being located on opposite sides of the country: **20** is in the southwest of Poland, close to the border with Germany, whereas **12** is found in the southeast, close to the border with Ukraine. Additionally, the highest number of statistically significant pairwise *F*_*ST*_ estimates was observed in clusters **20** (18 estimates) and **32** (17 estimates) (Supplementary Table S5).

**FIGURE 4:**
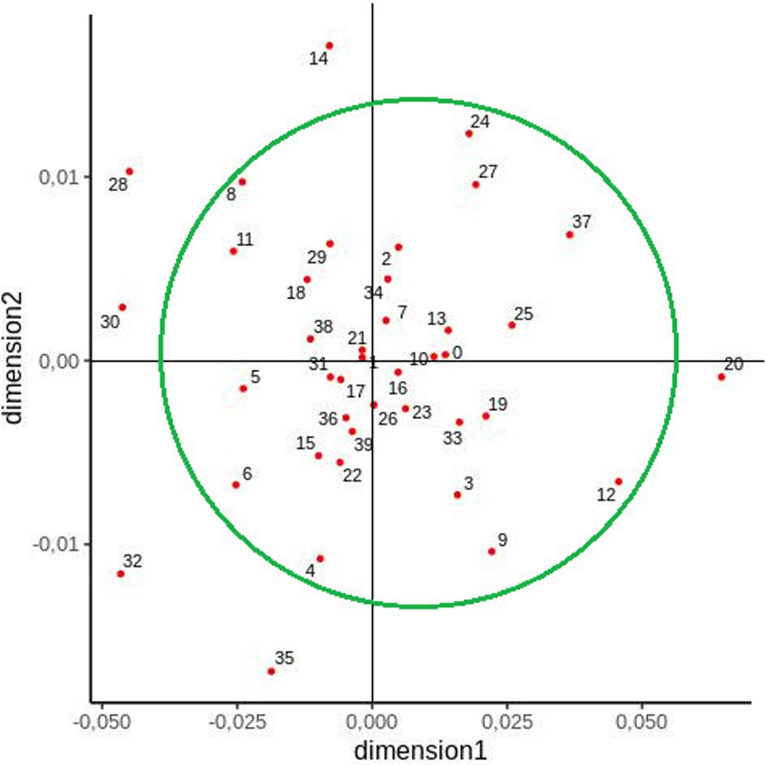
Two-dimensional MDS plot of cluster populations based on pairwise F_ST_ values.

Another MDS plot was constructed to visualize the relationships between generated clusters (Figure 4). In this case, a large group was formed including almost all clusters apart from the following: **12** (Bieszczady region), **14** (region of Słupsk), **20** (region of Jelenia Góra, Bolesławiec, and Zgorzelec), **28** (region of Wieluń, Częstochowa, and Lubliniec), **30** (Mazury region), **32** (region of Konin, Kalisz, and Ostrów Wielkopolski), and **35** (region of Włocławek and Kutno) (Figure 4).

### Analysis of Molecular Variance

Analysis of molecular variance analysis found that, for voivodeships, 99.25% of the variation was within the population and 0.75% among populations. Similar results were observed for the clusters: 98.73% of variation was within the population and 1.27% among populations. The Fixation Index was found to be 0.00746 for the voivodeships and 0.01269 for the clusters, with *p* = 0.00426, *p* = 0.01119, respectively (Table 2).

**TABLE 2:** Analysis of molecular variance (AMOVA) accounting for all voivodeships and clusters.

**Grouping method**	**Percentage of variation (%)**		**Fixation index (*F*_ST_)**
	**Among populations**	**Within populations**	
Voivodeships	0.75	99.25	0.007*
Clusters	1.27	98.73	0.01**

p-Values are indicated as *p = 0.00426, **p = 0.01119.

## Discussion

The genetic variability of the Y chromosome across the Polish population has been analyzed over the years, in studies in regard to different regions of Poland (Pepinski et al., 2001; Janica et al., 2005; Rebala and Szczerkowska, 2005; Soltyszewski et al., 2007; Wozniak et al., 2007; Wolanska-Nowak et al., 2009; Kostrzewa et al., 2013), among Lithuanian, Byelorussian, and Tatar minorities living in Poland (Pepinski et al., 2004c, 2005a; Janica et al., 2005) and in studies of larger population groups, including the entire population of the country (Lessig et al., 2001; Ploski et al., 2002; Kayser et al., 2005; Lessig et al., 2008; Soltyszewski et al., 2008; Rebala et al., 2013). Most of these studies were based on the PCR analysis of STRs. In contrast, the present study was performed using a microarray approach, which allowed the identification of several SNP on the Y chromosome; this approach yielded a detailed description of the genetic structure of the male population in Poland according to its voivodeships and counties and their clusters.

Haplogroup prediction was performed based on 496 SNP markers included in the Infinium HTS Human Core Exome microarray. Because the panel does not allow for differentiation of all possible haplogroups within the Eurasian metapopulation, only main haplogroups were considered for calculation of frequencies within specific voivodeships.

### Interpopulation Variability of Y Chromosome

For the sake of interpopulation analysis (including haplogroup frequencies from Slovakia, Slovenia, Czechia, Ukraine, Russia, Lithuania, Latvia, and Germany), we decided to use our results at the level that would allow for comparison with each country. The approach of different level of haplogroup estimation chosen for different types of analysis was also successfully applied by Altena et al. (2020).

Our results showed to be highly consistent with those obtained by Kayser et al. (2005), performed on a group of 913 Polish males. The frequency of R1a1^∗^ was almost exactly equal within both studies [57% in Kayser et al. (2005) and 56.93% in our sample]. Similarly, frequencies of haplogroups I^∗^ and R1b^∗^ were also comparable for both datasets (17.3 vs. 15.71 and 11.6 vs. 14.09% for hgs I and R1b, respectively). Because of lack of markers for hgs E3b^∗^ (M35) and N3^∗^ (M46) within the microarray used in the hereby presented study, we were not able to calculate exact frequencies of those hgs. Both of those are, however, subhaplogroups of hgs included in our results. It can be assumed that at least some part of hg E (3.84%) belongs to either E3b^∗^ (M35) [4.5% (Kayser et al., 2005)] or DE^∗^ (xE3b) (YAP) [0.5% (Kayser et al., 2005)], whereas the frequency of hg N (4.29%) is most probably a sum of N3^∗^ (M46) [3.7% (Kayser et al., 2005)] and K^∗^ (xN3, P) (M9) [0.5% (Kayser et al., 2005)]. The results’ concordance applies also to haplogroups with lower frequencies for the Polish population: J2^∗^ (M172) [2.5% (Kayser et al., 2005)] was predicted for 2.37% of samples, F^∗^ (xI, J2, K) (M89) [2.0% (Kayser et al., 2005)]—for 2.11% of the population, and P^∗^ (xR1a) (M74) [0.3% (Kayser et al., 2005)] for 0.26% of the population.

As an insight into the most recent Polish population, we performed a haplogroup prediction based on 496 27-Y-STR haplotypes published in 2017 by Spolnicka et al. (2017). A high level of similarity between both datasets is visible; however, lack of prediction for 140 samples (>25% of the studied sample set) seems to be the main reason for the inconsistencies found. One of those is the overrepresentation of haplogroup R1a [56.93 vs. 68.6%—haplogroup prediction based on Spolnicka et al. (2017)] and the remaining—the underrepresentation of hg I [15.71 vs. 6.8%—haplogroup prediction based on Spolnicka et al. (2017)]. The frequencies of some of the remaining haplogroups predicted (R1b, N, G, Q) are consistent with our findings. This bias clearly shows the necessity of using the biallelic markers for the purpose Y-chromosomal haplogroup determination.

While a part of both the Central and Eastern Europe and Baltic Rim Countries, Poland does vary from its neighboring countries in terms of the Y-chromosomal haplogroup structure at least at some level (for details, see Supplementary Table S6 with all national frequency data discussed below included). Results obtained in the hereby presented study are shown to be similar to the haplogroup frequencies of Slovenia (Zupan et al., 2013)—an Eastern Slavic country, and two countries considered as Western Slavic (Wozniak et al., 2010): the Czechia (Zastera et al., 2010) and Slovakia (Petrejcikova et al., 2010). The populations of those countries are considered homogenous (Rebala et al., 2007). This is especially the case for Poland and Czechia, as confirmed by the PCA of autosomal biallelic markers studied by Lao et al. (2008). In our case, the main difference between Slovenia, Czechia, Slovakia, and Poland laid in the frequency of hg R1a, found in almost 57% Polish males, whereas only between 36.9% (Slovenia) and 38% (Slovakia) for the aforementioned nations. Both Slovenia and Czechia are also characterized by a much higher level of hg R1b (20.3 and 24.8%, respectively), whereas for Slovakia the level of R1b seems similar to that of Poland (13.2 vs. 14.09%, respectively). Both Slovenians and Slovakians often fall within hg I (28.3 and 27.2%, respectively). Hg I is also frequently found in Czechia (20.1%), whereas in our results obtained for Poland its frequency is established at 15.7%. Hgs with lower frequencies, contributing to 12.57% of the Polish population (J, G, E, and N), are also found within all three of the aforementioned countries, the only exception being haplogroup N, not present in the Slovenian population. Those haplogroups sum up to 12.2, 17.2, and 17.4% of Slovenian, Slovakian, and Czechia populations, respectively.

The populations of Lithuania (Kasperaviciute et al., 2004) and Latvia (Pliss et al., 2015) seem genetically more distant from Poland, regardless of the Polish-Lithuanian Union that lasted for more than 400 years between the XIV and XVIII century (Ploski et al., 2002). In both of those countries, hg N is one of the two most commonly found haplogroups (36.7 and 41.5%, respectively), present only in 4.29% of Polish population, with the other most frequent hg being R1a (44.9 and 37.8%, respectively). R1a is the most common haplogroup in Poland, found in almost 57% of the population. The Germanic R1b haplogroup is found in Latvia and Lithuania on a much lower level than in Poland, understandably (Wozniak et al., 2010). For Lithuania its frequency is estimated to be below 5.1% [as (Kasperaviciute et al., 2004) did not differentiate between R1b and Q, this is the sum of both] and for Latvia—7.6%, which is almost three and two times less than what can be found in Poland, respectively.

As Maliarczuk and Derenko (2008) investigated levels of haplogroup frequencies through the European part of Russia, some conclusions can be drawn regarding their similarity and differences to the population of Poland, also in comparison to the in-between Ukraine (Mielnik-Sikorska et al., 2013b). For both Russia and Ukraine, hg R1a is still common [Northern Russia (NR)—34.2%, CR (Central Russia)—46.54%, South Russia (SR)—55.4%, Ukraine—43.9%]; however, in NR, hg N is the most frequent one (43% of the population). For CR and SR, the value of haplogroup N frequency is lower (17.2 and 10%, respectively), yet much higher than for Poland (4.29%). Haplogroup N was not found by Mielnik-Sikorska et al. (2013b) within the Ukrainian population. Similarly to Lithuania and Latvia, both Russia and Ukraine are much lower in R1b subhaplogroup than Poland (Ukraine and NR—5.4%, CR—7.1%, SR—4,8%). Haplogroup I is found with a high frequency in Ukraine and SR (28.4 and 21%, respectively) and CR and NR (17.5 and 13.1%, respectively), unlike in Poland, where we calculated it can be found in greater than 6% of the population. In all of the aforementioned countries, haplogroup J is found in less than 5% of the population (Ukraine—3.4%, NR—1.8%, CR—4.0%, SR—3.5%), much like in Poland (3.22%). Furthermore, it is the J2 subhaplogroup that is found more frequently, including Ukraine, where J2 is found almost exclusively.

As expected, from all of the neighboring countries, Germany is the one most distant from Poland in Y-haplogroup distribution. As observed by Kayser et al. (2005), the frequency of R1b is almost three times higher for Germany than for Poland (38.9 vs. 14.09%), the frequency of I—almost four times (23.6 vs. 6.02%), whereas R1a is found almost three times less frequently in Germany than in Poland (17.9 vs. 56.93%, respectively).

### Intrapopulation Variability of Y Chromosome

Y-chromosome polymorphism analysis and both Y-SNP and Y-STR typing indicate that the Polish population is highly homogeneous both in terms of the entire country (Ploski et al., 2002) and separate regions (Pepinski et al., 2004a; Soltyszewski et al., 2007; Wozniak et al., 2007; Wolanska-Nowak et al., 2009). While the present study generally confirmed this result, it also allowed a more detailed insight at the diversity of the Polish population at the level of administrative units and clustered regions: the genetic information was related to place of residence, with participants from all voivodeships and the majority of counties; further testing was also facilitated by the use of clustering as an additional method of population grouping. A goal of the study was to see if a different result could be achieved by using a large set of data; examining a well-established representation of the entire Polish population and the use of regional clustering, we will get different result. Our findings indicate homogeneity with most variation occurring within populations at the voivodeship and cluster level: 99.25% for voivodeships and 98.73% for clusters. Only a small proportion of total variance was attributed to variation among groups in voivodeships (0.75%) and clusters (1.27%). This observation is consistent with Kayser et al. (2005), who reported 0.3% variability computed for Y chromosome SNPs.

The observed differences between the studies can be accounted for by differences in sample population number and profile. The present study was based on a data set comprising 2,705 individuals from all 16 voivodeships and 337 of the 380 counties, whereas the results of Kayser et al. (2005) were probably based on inhabitants of the selected cities in Poland (Wrocław, Warsaw, Lublin, Kraków, Bydgoszcz, Gdańsk, Szczecin, and Suwałki). Unfortunately, because of a lack of such studies, it is not possible to perform a detailed comparison of haplogroup frequencies for all voivodeships and counties.

Regarding the numbers of different haplogroups in voivodeships, the present findings correspond with the variability of mtDNA in the Polish population (Jarczak et al., 2019). In the earlier study, the Silesia voivodeship was indicated as the region with the greatest number of mtDNA haplogroups (19 of 21). A similar situation is observed in the present study: 10 of 11 total Y-chromosome haplogroups were found in individuals from Silesia. In contrast, Holy Cross voivodeship demonstrated the least variety, with only 10 mtDNA haplogroups. The differences shown in the present study are not so highlighted, with most voivodeships being characterized by six or seven haplogroups. The distribution and the frequency of haplogroups indicate that the Polish population is characterized by greater diversity in the case of mtDNA (Jarczak et al., 2019); several haplogroups were found to be present in the Polish population, with hg H demonstrating the highest frequency. Furthermore, four hgs (H, U, J, T) accounted for 82.38% of the studied population; however, many others prevalent in the European population (K, W, I, HV, V) were also observed. The Y-chromosome SNP analysis found R to be present in more than 71% of Polish males and, together with hg I, represents the vast majority of Y chromosome haplogroups (86.73%).

In contrast to previous studies, the present study examined a larger number of samples taken from individuals from all administrative regions of Poland and applied clustering as an additional method of grouping the populations. However, slight differences were observed between some studied regions according to the method of analysis. The Lodz voivodeship, for example, was found to be distinct from other voivodeships with regard to mtDNA variability (Jarczak et al., 2019). The historical basis for this variation is unclear: in contrast to West Pomerania and Warmia–Mazuria, Łódź, as a native voivodeship (excluding west part—see below), has not been the site of large-scale migration. Furthermore, MDS visualization indicated that almost all clusters were grouped together, indicating population homogeneity; however, clusters **12** (Bieszczady region), **14** (Słupsk region), **20** (Jelenia Góra, Bolesławiec, and Zgorzelec region), **28** (Wieluń, Częstochowa, and Lubliniec region), **30** (Mazury region), **32** (Konin, Kalisz, and Ostrów Wielkopolski region), and **35** (Włocławek and Kutno region) were distinct from this grouping, suggesting that genetic differences exist between their inhabitants.

The Bieszczady region, for example, is located in the southeastern part of Poland and is considered geographically distant from the rest of the country. It is characterized by one of the highest levels of forest cover in Poland and a lack of large urban centers. Furthermore, the region was historically affected by mass displacement of Lemkos and Ukrainians, with about 700,000 people having been displaced from the former Rzeszów voivodeship, particularly the counties of Lesko, Przemyśl, and Sanok: the Ukrainian people were moved to the east, whereas the Lemkos mainly settled the Lower Silesia and Masuria, which were granted to Poland after WWII. The Bieszczady region itself was resettled from the late 1950s (Ociepka, 2001).

Cluster **30**, which corresponds to the Mazury region, has a different history to Bieszczady but was also a site of mass resettlement. Before the WWII, the region was part of German East Prussia; however, from 1946 to the 1970s, the Masurians inhabitants migrated to Germany and were replaced by people from other regions of Poland, such as those resettled from the Bieszczady region.

In the case of clusters **20**, **28**, **30**, and **32**, however, the historical explanation for their separation based on demographic processes is unclear. There are some historical justifications, such as the complete removal of at least 250,000 native Polish citizens, and their replacement by German citizens mostly from the Baltic region, i.e., the Reich District Land of the Warta river (Ger. *Der Reichsgau Wartheland*) (Eberhardt, 2000). The Warta river land covered a vast area from Poznań in the west, through the Kalisz region to Lodz in the east, and reaching as far as Inowrocław in the north, which more or less corresponds to the areas covered by cluster no. **32**.

Interestingly, while previous analyses based on mtDNA variability (Jarczak et al., 2019) generally identify different regions as being genetically distinct, some similarities between the studies are visible. The region of Western Kuyavia (cluster no. 47 in the cited study) seems to be comparable to cluster **32**, at least in some counties, in that it was also found to be genetically distinct. In addition, the previous study based on mtDNA variation indicated the Mazuria region (cluster no. 49 in the cited study) to be genetically distinct, and the present study found its analogous cluster to be the same (no. **30**). However, it is not possible to make a full and accurate comparison between the two studies because of different number of clusters.

The interpolation maps were used to visualize regional differences between observed frequencies of hgs in Poland. As shown in Figure 1, haplogroup R1a is distributed mostly in the center part of Poland with a few regions on the west and east of the country. Interestingly, R1a was also found to be present in high numbers in eastern regions, including the Podlaskie and Warmian–Mazurian voivodeships, as well as almost all of the Lublin voivodeship; similar results were also obtained from central regions and Western Pomerania, which may have some historical basis. In contrast, R1b was more widely distributed, reaching farther east and west than the others; however, it is observed at relatively low frequencies in regions adjacent to the western and eastern borders of Poland. Such a pattern of distribution of hg R in the Polish population can reflect some historical events such as massive human migrations or the changes in the territorial borders.

A similar situation was observed in the case of hg I, whose distribution also followed geographic lines and possibly historical events. Haplogroup I is found to be represented mostly in western Poland and some region of eastern Poland, mostly in the Podlaskie and Lublin voivodeships, but also reaches the eastern parts of Mazovia, the western parts of Warmian–Mazurian, and almost all of Subcarpathia, which makes these regions similar to the west in terms of haplogroup frequency.

Interestingly, in the case of hg N, the Podlaskie voivodeship is distinct from the remaining voivodeships: as it was mentioned above, the frequency of hg N, which is common among the populations of Lithuania (Kasperaviciute et al., 2004) and Latvia (Pliss et al., 2015) and other inhabitants of northeast Europe, is 14.55% in this area and brings Podlaskie closer to the northern regions in this regard. In contrast, hg E displays much greater homogeneity across the map, with fewer marked differences between regions.

The comprehensive analysis of Y-chromosome variability described in the present study, i.e., based on the data from 2,705 individuals, including those from all voivodeships and most counties, and employing clustering as an additional method of population grouping, is the first of its type to be performed on the population of Poland. The findings confirm that the Polish population is characterized by a high degree of homogeneity, with only slight genetic differences being observed at the regional level. The use of regional clustering as an alternative to counties and voivodeships provided a more detailed view of the genetic structure of the population; the cluster analysis also identified any misleading differences observed between voivodeships.

Such a broad genetic analysis of Polish population should be able to give insights into the history of different regions of the country, especially given the individuals studied were asked to include information concerning their ancestry. The quality of answers given was, however, less than satisfactory, and so no conclusions can be drawn, because the history of the paternal line of those people remains unknown. It seems the only way to pursue the search for local history is to study populations with regard to even three-generations-down worth of genealogy knowledge, as shown by Rebala et al. (2013).

The results of the present study, together with previously published data about mtDNA variability, could serve as the basis for the further research into the connection between the modern and ancient times of Poland with regard to human migration and resettlement, as well as historical and cultural influences. Furthermore, regional differences identified by the mtDNA variability study and the present one highlight the need for additional division of the population by cultural and ethnic criteria in such studies rather than just by geographical or administrative regionalization. Representatives of ethnic (Karaites, Tatars), cultural (Kashubians, Kurpie, Podhale highlanders), and indigenous groups in specific regions of Poland should be included in future analyses.

## Data Availability Statement

The datasets presented in this study can be found in online repositories. The names of the repository/repositories and accession number(s) can be found below: https://ega-archive.org/studies/EGAS00001004111.

## Ethics Statement

The studies involving human participants were reviewed and approved by University of Lodz Ethics Review Board. The patients/participants provided their written informed consent to participate in this study.

## Author Contributions

DS conceptualized and supervised the study, provided the funding, organized and integrated the data. BM provided the funding, organized and integrated the data. ŁG, JJ, and MU performed bioinformatic analyses. MS-K and MSł performed microarray analysis. DS, JJ, WL, ŁG, PB, MSł, MS-K, MU, MSz, and AO analyzed the result of differences in haplogroups frequencies within Polish population. JJ, MSł, ŁG, WL, PB, AO, MSz, and DS drafted the manuscript. All authors contributed to the article and approved the submitted version.

## Conflict of Interest

The authors declare that the research was conducted in the absence of any commercial or financial relationships that could be construed as a potential conflict of interest.

## Appendix A: Supplemental Data

### Web Resources

BBMRI-ERIC Directory, https://directory.bbmri-eric.eu/

Python, https://www.python.org/

Scikit-learn, https://scikit-learn.org/sta

QGIS, http://qgis.org

Geodesic and Cartographic Documentation Center, https://gis-support.com/spatial-datasets-for-poland/

Google Maps Api, https://developers.google.com/maps

GenomeStudio, https://www.illumina.com/techniques/microarrays/array-data-analysis-experimental-design/genomestudio.html

StrandScript, https://github.com/seasky002002/Strandscript

European Genotype Archive, https://www.ebi.ac.uk/ega/

yHaplo, https://github.com/23andMe/yhaplo

International Society of Genetic Genealogy. Y-DNA Haplogroup Tree 2016, http://www.isogg.org/tree/

Arlequin, http://cmpg.unibe.ch/software/arlequin35/

R, https://www.r-project.org/.
