# Supplementary material for: Y-Chromosome Genetic Analysis of Modern Polish Population
Source: Front Genet. 2020 Oct 23;11:567309. doi: 10.3389/fgene.2020.567309 (PMC7644898; doi:10.3389/fgene.2020.567309)

**The list of supplementary materials:**

**Tables:**

**Table S1.** The list of clusters containing the information about the cluster number to which appropriate county as well as the name of geographic region were assigned.

**Table S2.** The list of markers according to HGVS recommendations for the description of sequence variants.

**Table S3.** The list of typed haplogroup for each sample.

**Table S4.** F_ST_ values calculated between each pair of voivodeships.

Statistically significant results are marked with plus (+) (P-values < 0.05, based on 10 000 permutations).

**Table S5.** F_ST_ values calculated between each pair of clusters.

Statistically significant results are marked with plus (+) (P-values < 0.05, based on 10 000 permutations).

**Table S6.** Haplogroup prediction and the comparisons of theirs frequencies in the neighbouring populations (Czech Republic, Germany, Latvia, Lithuania, Russia, Slovakia, Slovenia, Ukraine).

**Figures:**

**Supplementary Figure S1.** The main directions of official resettlement of Polish citizens in years 1944-1948 and 1955-1959 from territory lost to the Soviet Union to lands gained at the expense of Germany (data based on Czerniakiewicz J., Repatriation of the polish population from USSR 1944-1948, PWN, Warsaw 1987; in Polish: Repatriacja ludności polskiej z ZSRR 1944–1948, PWN, Warszawa 1987)


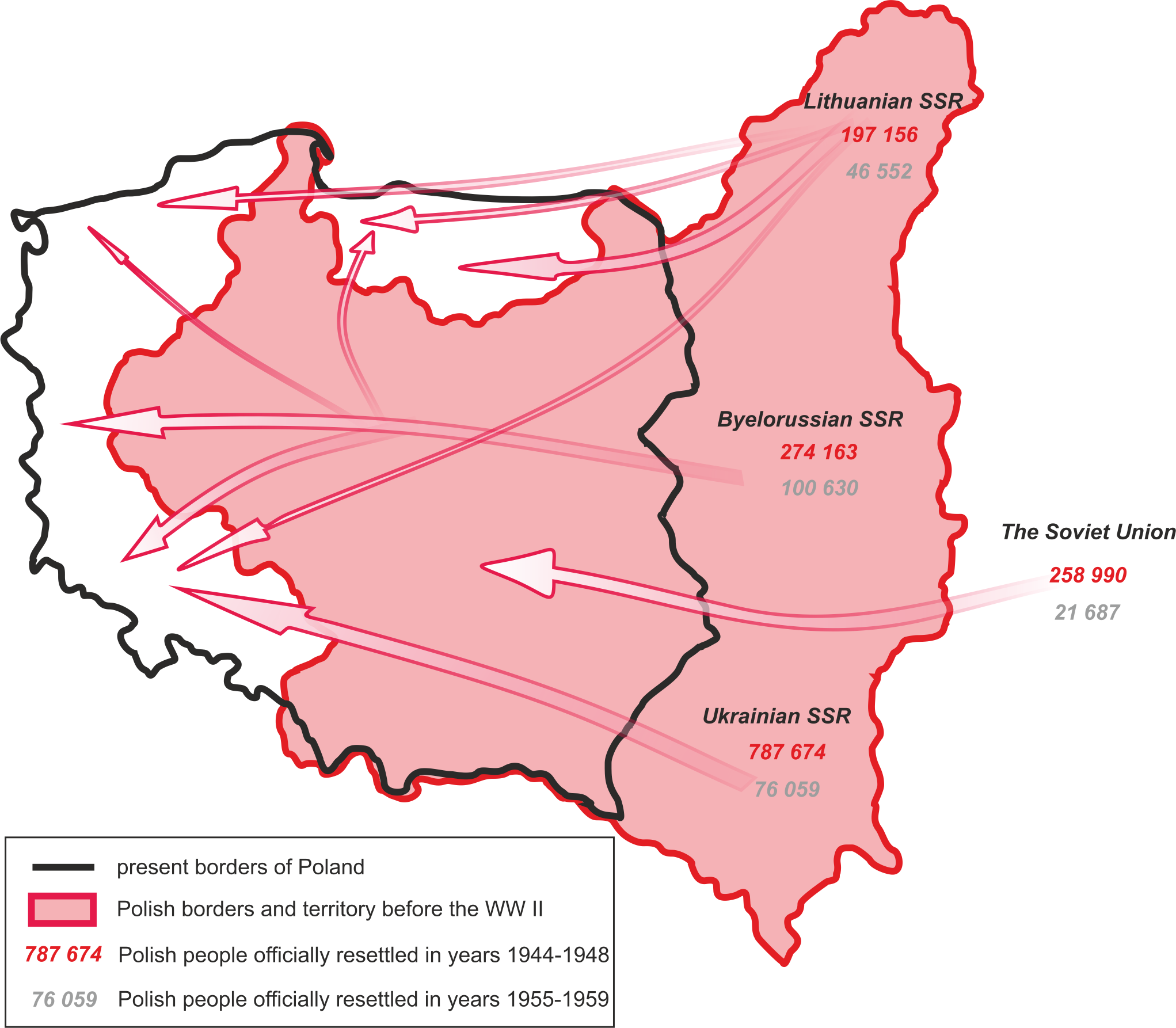


**Supplementary Figure S2.** The most important mass migrations in years 1944-1949 on territory covering lands belonging before and after the war to Poland. Estimated data based on:


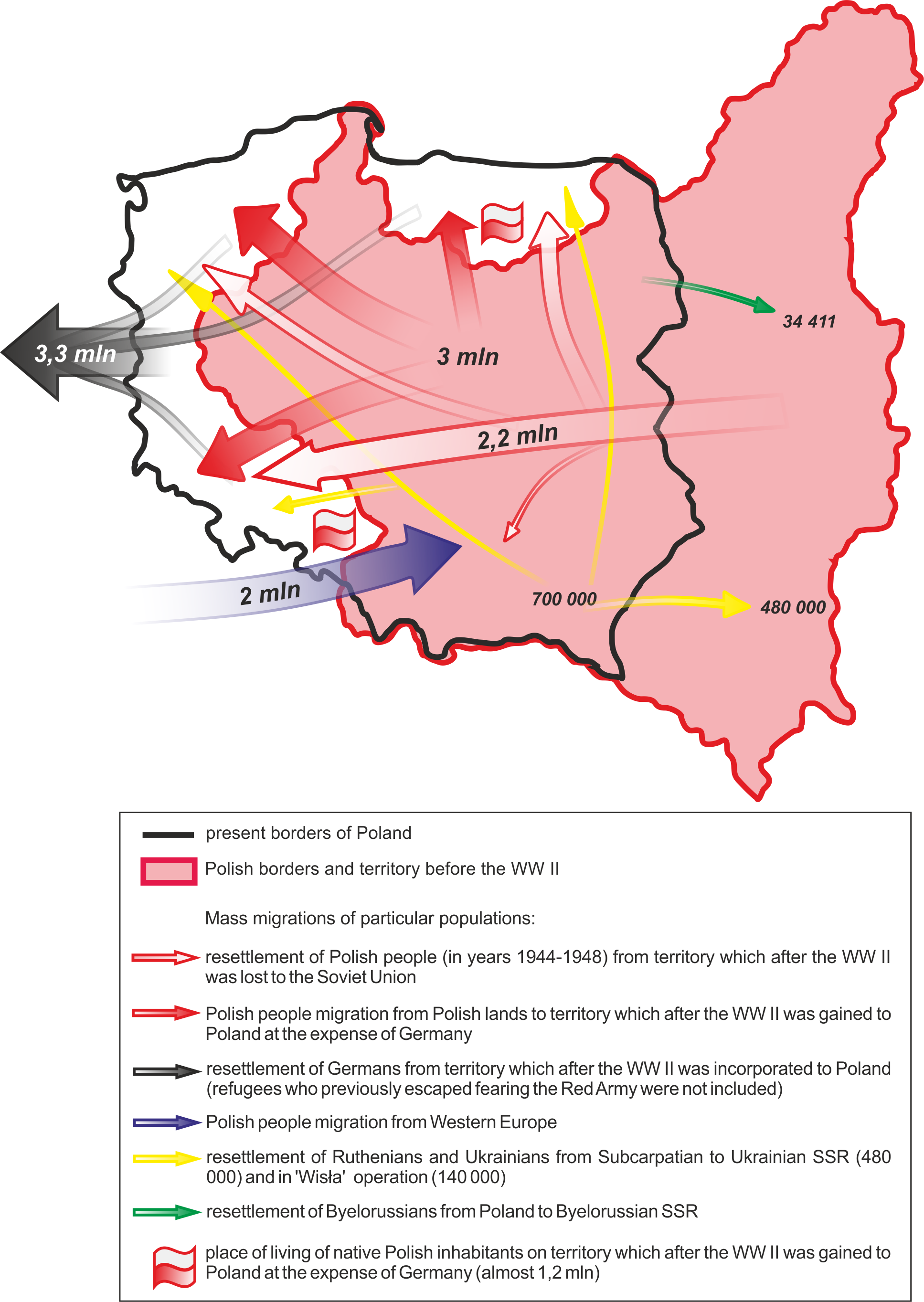


**Supplementary Figure S3.** Studied counties grouped into clusters.

Counties are grouped with color to represent cluster, numbers stand for cluster ID. White color means no data.


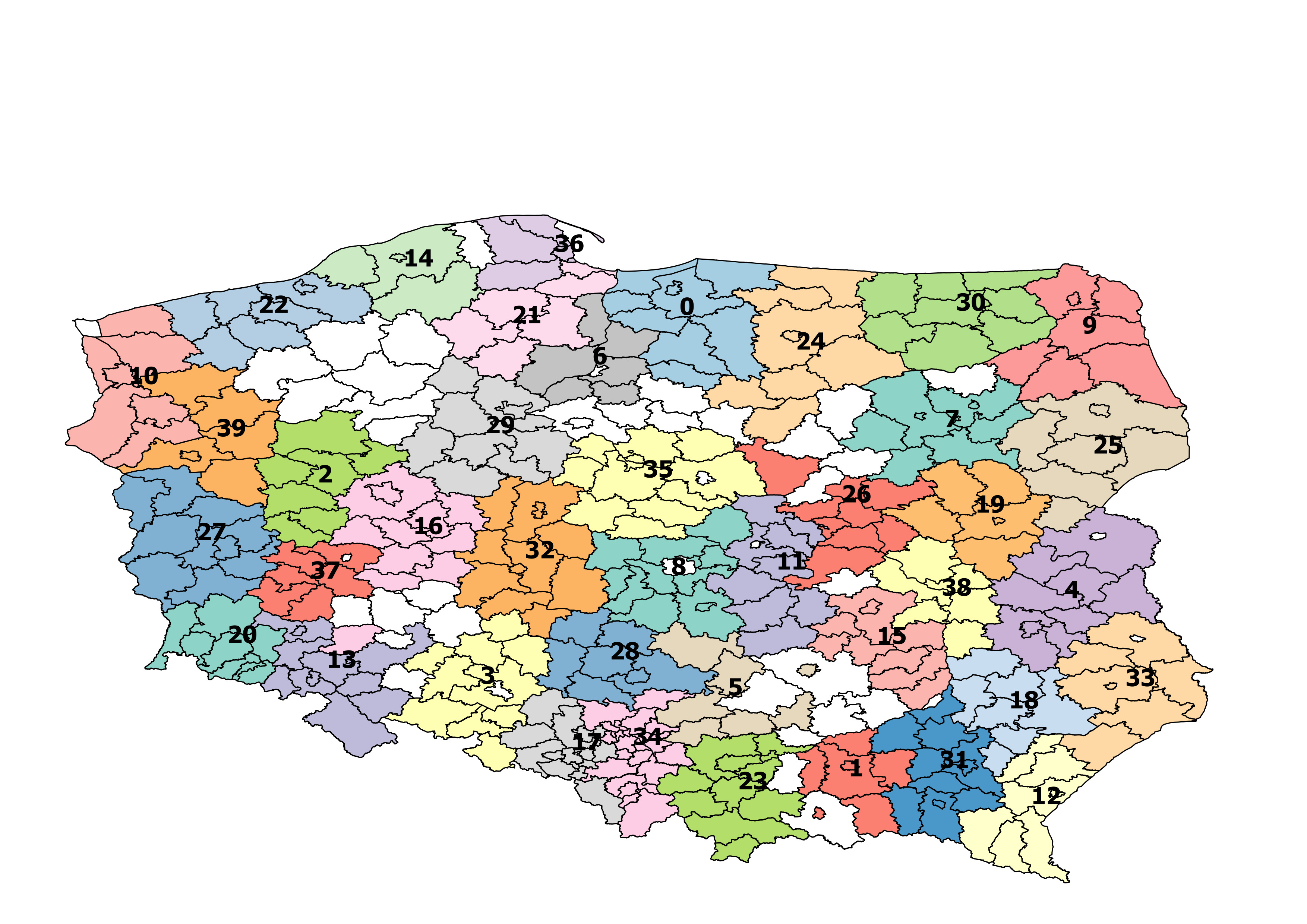


**Supplementary Figure S4.** Matrix of pairwise F_ST_ genetic distances calculated between each pair of voivodeships based on the Y-chromosome SNPs.


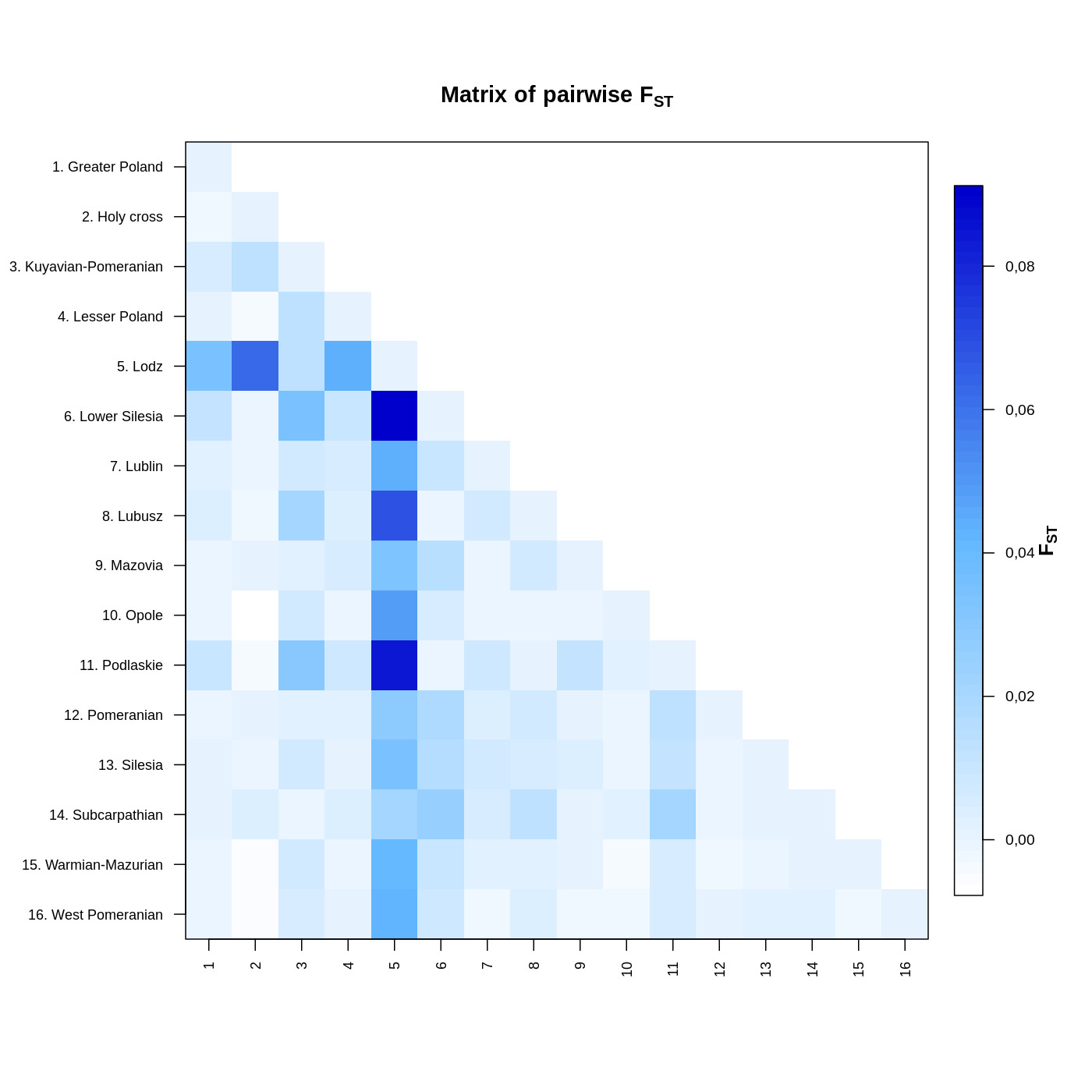


**Supplementary Figure S5.** Matrix of pairwise F_ST_ genetic distances calculated between each pair of clusters based on the Y-chromosome SNPs.


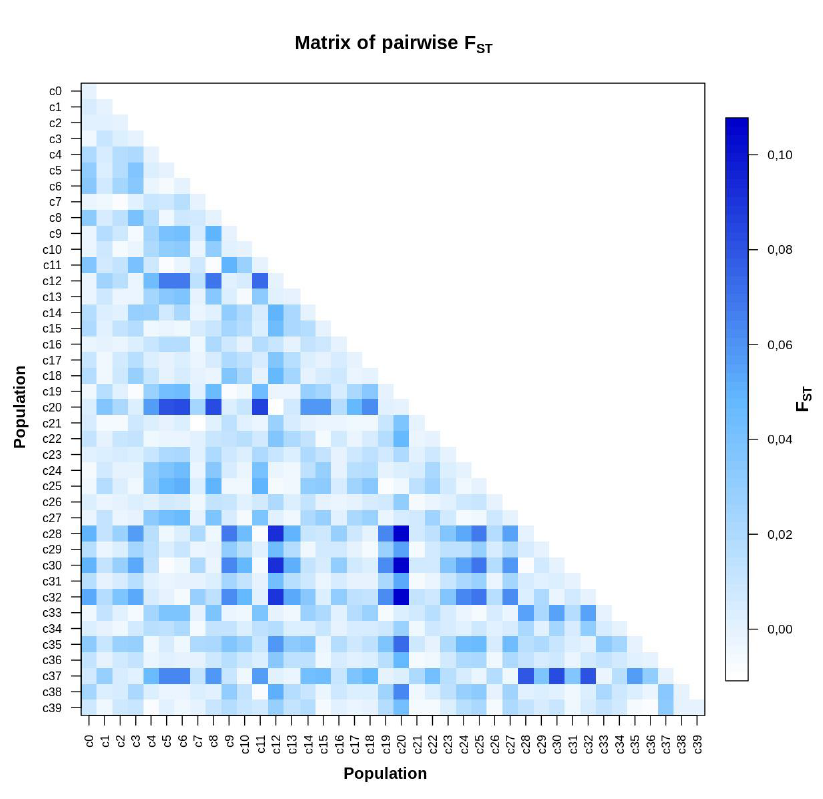

Supplement: Supplementary file 1 [file Data_Sheet_1.docx]
